# Exploring the Potential of Roselle Calyx and Sappan Heartwood Extracts as Natural Colorants in Poly(butylene Succinate) for Biodegradable Packaging Films

**DOI:** 10.3390/polym15204193

**Published:** 2023-10-23

**Authors:** Wordpools Nansu, Sukunya Ross, Amonrut Waisarikit, Gareth M. Ross, Pensri Charoensit, Nungruthai Suphrom, Sararat Mahasaranon

**Affiliations:** 1 Department of Chemistry, Faculty of Science and Centre of Excellence in Biomaterials, Naresuan University, Phitsanulok 65000, Thailand; worrapholn61@nu.ac.th (W.N.); sukunyaj@nu.ac.th (S.R.); amonrutw62@nu.ac.th (A.W.); gareth@nu.ac.th (G.M.R.); nungruthais@nu.ac.th (N.S.); 2 Faculty of Pharmaceutical Science and Center of Excellence for Innovation in Chemistry, Naresuan University, Phitsanulok 65000, Thailand; pensric@nu.ac.th

**Keywords:** biodegradable polymers, PBS film, food packaging, roselle, sappan heartwood, mechanical properties

## Abstract

Recently, there has been a growing concern among consumers regarding the safety of packaging products, particularly due to the presence of potentially harmful substances like synthetic pigments and inorganic dyes. These substances, which are often used to attract consumer attention, can migrate and contaminate products over extended shelf storage periods. To address this issue, the focus of this research was the development of a biodegradable packaging film using poly(butylene succinate) (PBS) incorporated with natural colorants extracted from roselle (RS) and sappan heartwood (SP). RS and SP serve as non-toxic and alternative pigments when compared to synthetic colorants. The biodegradable packaging films were prepared using blown film extrusion, encompassing different weight percentages of RS and SP (0.1%, 0.2%, and 0.3%). The films exhibited distinct colors, with RS films appearing pink to purple and SP films exhibiting an orange hue. The water vapor transmission rate slightly decreased with an increasing content of RS and SP extracts, indicating improved barrier properties. Additionally, the films showed reduced light transmittance, as evidenced by the UV–Vis light barrier results. The degree of crystallinity in the films was enhanced, as confirmed by X-ray diffraction and differential scanning calorimetry techniques. Regarding mechanical properties, the PBS/RS and PBS/SP films exhibited slight increases in tensile strength and elongation compared to neat PBS films. Moreover, the blended films demonstrated higher stability after undergoing an aging test, further highlighting their potential for use in biodegradable packaging applications. The key advantages of these films lie in their non-toxicity, biodegradability, and overall environmental friendliness.

## 1. Introduction

Nowadays, plastic packaging products are widely used in many applications for protection, transportation, storage, protection, quality, and safety. The demand for and interest in packaging plastics are expected to increase because of online shopping and E-commerce growth that is projected to rise at 11.2%. For example, food packaging products are expected to have used ~320 million tons of plastic in 2022 [1].

Generally, commercial packaging products made from non-degradable petroleum-based polymers cause environmental problems such as toxic chemical release and decomposition into microbeads and microplastics [2,3]. During plastic processing, chemical additives (such as plasticizing agents, antioxidant agents, light and thermal stability agents, antistatic agents, nanoparticles, and dye pigments) are loaded into the plastic, and these additives are able to migrate out of the plastic before, during, and after use and contaminate the products/environment [4,5].

Numerous studies have documented the exploration of alternative materials, particularly bioplastic materials, as substitutes for synthetic and non-biodegradable petroleum-based polymers. Among bioplastic materials used for biodegradable packaging, poly(lactic acid) (PLA) [6,7,8,9], poly(hydroxy butyrate) (PHB) [10], poly(butylene succinate) (PBS) [11], poly(ethylene oxide) (PEO) [12], polycaprolactone (PCL) [13], polyhydroxyalkanoates (PHA) [14], and poly(butylene adipate terephthalate) (PBAT) [15] are reported to be biodegradable and biocompatible polymers.

In addition to biodegradable materials, natural colorants have been investigated as additives to enhance the product color, pricing, light barrier properties, plasticizing ability, antioxidant and antimicrobial activities, as well as product shelf life, thereby serving as potential replacements for toxic pigments derived from organic or inorganic sources [7,16,17]. Among the various sources, natural colorants derived from plant resources, including seeds, barks, leaves, flowers, fruits, calyx, heartwoods, and peels, have gained significant popularity and widespread usage, surpassing other options such as minerals and animals [18,19,20]. 

The natural colorants sourced from the heartwood of *Caesalpinia sappan* L. (*sappan*, SP) and roselle calyx of *Hibiscus sabdariffa* L. (*Roselle*, RS) contain flavonoids and anthocyanins as the main constituents responsible for coloring [21,22]. SP, a traditional plant, finds broad applications in various domains, including traditional medicines, foods, beverages, pharmaceuticals, dyes, and cosmetics [23,24]. It comprises brazilein and brazilin as the main chemical compounds that give the red color to the extract [25]. These chemical compounds exhibit low toxicity, and their color can be altered through chemical structure transformations. RS is a local plant in Thailand and produces anthocyanins that present with pH sensitivity [19,26,27,28]. Furthermore, when incorporated into products such as food, beverages, and packaging, these natural colorants have demonstrated the ability to prevent oxidative damage [4,29,30].

In our previous works, we fabricated composite films from PLA mixed with lemongrass leaf powder [9]. The lemongrass powder showed a green color shade and influenced the composite’s properties. Another study focused on the development of a composite packaging film composed of PLA integrated with spent coffee grounds (SCG) [6,8]. SCG was also observed to slightly improve the mechanical properties of films, with a dark brown appearance. Nevertheless, an inadequate distribution and dispersion of lemongrass and SCG powders within the PLA film phase were observed. 

In this study, the focus was on utilizing natural colorants in the form of solutions derived from SP and RS. This approach aimed to overcome the limitations associated with natural colorants in powder form and to investigate their potential synergistic effects on the physical and mechanical properties of biodegradable films. 

PBS was adopted as the biodegradable polyester instead of PLA due to its performance as a ductile polymer (glass transition and melting temperature of approximately −35 °C and 114 °C, respectively), and it exhibits much higher elongation at break than PLA [31]. In addition, PBS also has excellent processability, good thermal stability, softness, and low gas barrier properties that are suitable for extruded and blown products [32].

Therefore, the purpose of this research was to prepare biodegradable packaging films based on PBS. PBS and either RS or SP extracts were mixed and processed by a twin-screw extruder to produce polymer pellets before being blown into films by a blown-film extruder. All process parameters were carefully adjusted to create favorable conditions for achieving a uniform dispersion and distribution of the added component into the films. 

The physical, chemical, mechanical, and thermal properties, including the stability of the films, were characterized. These films hold great potential for biodegradable packaging applications, as they are environmentally friendly and do not release harmful chemicals. 

## 2. Materials and Methods

### 2.1. Materials

The flower of *Hibiscus sabdariffa* L. and the wood stalk of *Caesalpinia sappan* L. were purchased from a local market in Phitsanulok province (Osot-Niyom shop), Thailand. These materials were subsequently subjected to extraction processes to yield the roselle calyx (RS) and sappan heartwood (SP) extracts. 

Polybutylene succinate (PBS) was purchased from BC Polymers Marketing Co., Ltd. (Bangkok, Thailand) (grade FD92PM) with a specific gravity of 1.24 g/cm^3^, Mw > 200,000, MFR of 4 g/10 min and T_m_ of 84 °C. A commercial grade of 95% ethanol for natural colorant extraction was supplied by TKK Science Company (Bangkok, Thailand). 

### 2.2. Extraction of Natural Colorants 

Sappan heartwood (SP) was cut by a wood chipper to obtain small pieces of approximately 5 × 50 mm in size, then 50 g of SP was macerated with 300 mL of 80% ethanol at room temperature for 72 h without light contact before filtration by filter paper No 40. Then, the solvent was further evaporated by a rotary evaporator at 60 °C, 100 rpm to give the viscous crude extract with a yield of 7% *w*/*w*. Finally, the 20% *w*/*v* solution of the viscous SP extract was prepared by dissolving a certain amount of extract in 95% ethanol and it was kept at a controlled temperature of 4 °C before being used [33]. 

For the extraction of roselle calyx (RS), the size of the dried RS was reduced by a blender and filtered by a 60-mesh sieve. Then, 10 g of RS powder was extracted by 150 mL of 80% ethanol and maintained at 50 °C for 1 h. The RS extracted solution was filtered and evaporated by a rotary evaporator at 60 °C and 100 rpm to give the crude RS extract with a yield of 7% *w*/*w*. After that, a 20% *w*/*v* solution of viscous crude RS extract (in 95% ethanol) was prepared as a ready-to-use extract and stored at 4 °C without light contact to prevent any degradation of the extract [26,34]. 

The freshly prepared extracts were carefully characterized utilizing thin-layer chromatography and ultraviolet spectroscopy methodologies, with the aim of establishing baseline control data. Furthermore, a comparative analysis was undertaken to evaluate the stability of the stored RS and SP extracts, wherein their attributes were contrasted with those of newly prepared extracts preceding their blending with PBS.

### 2.3. Processing of Biodegradable Films

Biodegradable films were fabricated using a twin-screw extruder (LABTECH Model LTE 16-40, LabTech Engineering Company Ltd., Samut Prakan, Thailand) for compounding PBS with natural colorant extracts (SP and RS), followed by a blown-film extruder (LABTECH Models LE20-30/C and LF-250, Thailand) for film formation. Different amounts of either SP or RS were used at 0.1, 0.2, and 0.3% wt. The extrusion conditions for compounding were set at the temperature of 90–100 °C (feeding zone), 100–110 °C (compression zone), and 120 °C (metering zone) for complete melting without plastic deformation. The blown-film extrusion profile temperatures were set at 100, 100, 110, 110, and 120 °C, from the feeding zone to the die zone, respectively. Film thickness was controlled at 70 µm. Films were kept at room temperature without moisture and light contact for 7 days before characterization. 

### 2.4. Characterizations

#### 2.4.1. Color Parameter Index

The film color parameter index was determined using a color reader CR-20. Measurements were taken at five different areas per sample, and the resulting data were averaged to obtain values for lightness (*L*), redness (*a*), yellowness (*b*), and ∆*E* (color difference) using Equation (1). Films were kept at room temperature for 7 days (original) and 2 months for the stability aging test before measurement of the color parameter index.

(1) $${∆E^{*}=\left[{(L}_{1}^{*}-L_{2}^{*}\right)}^{2}+{\left(a_{1}^{*}-a_{2}^{*}\right)}^{2}+{\left(b_{1}^{*}-b_{2}^{*}\right)}^{2}{]}^{1/2}$$

where $L_{1}^{*}$ refers to the L* value of a sample, $L_{2}^{*}$ refers to the L* value of the reference, and similarly for the other two parameters. In the case of the stability aging test, the reference was the same film at its origin.

#### 2.4.2. Water Vapor Transmission Rate (*WVTR*)

The water vapor transmission rate (*WVTR*) of the biodegradable packaging films was tested by the gravimetric method followed by ASTM E96-80 [35]. The samples with a size of 5 × 5 cm were prepared and sealed in a glass jar with anhydrous silica gel pellets. The samples were weighed at time intervals. Films were kept at a controlled room temperature with the humidity at 50% in a desiccator. The weight change was collected every day for one month and calculated by the following Equation (2):

(2) $$WVTR=\frac{W}{AxTime}\;\;(\frac{g}{m^{2}}\cdot day)$$

where *W* is the increased weight of the film sealed in the glass jar (g), *A* is the area (m^2^), and *Time* is the day of testing (day).

#### 2.4.3. Light Transmittance

Light transmittance of the films was measured by a UV/Vis spectrophotometer (model SPECORD 200 PLUS, Analytik Jena, Jena, Germany). Square-shaped films with dimensions of approximately 50 × 50 mm and a controlled thickness of 70 µm were prepared. The films were then stored at room temperature for 7 days (considered the original condition) and subjected to an aging test at room temperature for 2 months. Spectral measurements were performed at room temperature, with the wavelength range set from 200 to 800 nm. 

#### 2.4.4. Stability

Film stability was tested by an accelerated aging test. The film samples were kept at room temperature in 50% humidity for 2 months before testing the color parameter index, light transmission by UV–VIS, and mechanical properties. 

#### 2.4.5. Surface Morphology

The surface morphology of the films was analyzed by scanning electron microscopy (SEM) using a Leo1455VP model (1–30 keV). The samples were cut into small pieces and fixed on two layers of tape and coated with a thin layer of gold before testing. 

#### 2.4.6. Functional Groups

The functional groups of the natural colorant extracts and films were measured by an Attenuated Total Relevance Infrared Spectrometer (FT-IR ATR mode, Perkin Elmer (Waltham, MA, USA), Model Spectrum GX, 4 cm^−1^ resolution). The wavelengths were analyzed in the ranges of 4000–400 cm^−1^.

#### 2.4.7. Crystallinity

The crystallinity of the films was characterized by the X-ray diffraction (XRD) technique (with an X-ray Diffractometer (XRD) BRUKER (Billerica, MA, USA), D2 Phase, software: Diffrac EVA v.5.0r). Samples were prepared in a square shape of 50 × 50 mm and put into the sample holder. The 2θ detection angle was collected from 5° to 80°, and the crystallinity was calculated following Bragg’s law equation.

#### 2.4.8. Tensile Properties

The tensile properties of the films were measured with a Universal Testing Machine (INSTRON^®^ CALIBRATION LAB model 5965, software: Instron Bluehill v3.73.4823, Bluehill Universal, Norwood, MA, USA) with ASTM D-882. Films (10 pieces/sample) were cut into dumbbell shapes and kept at room temperature for 7 days before age testing for 2 months. The test parameters were a gauge length of 57 mm, cross ahead speed of 200 mm/min, load cell of 1 kN, and load force of 100 N. Films were tested in two directions, the machine direction (MD) and transverse direction (TD). Tensile strength at break, % elongation at break, and the modulus were studied using the following equations [36]:

(3) $$Tensile\;strength\;at\;break\;(\mathrm{Mpa})=\frac{Force\;at\;break\;(N)}{Area\;of\;specimen\;(m^{2})}$$

(4) $$\%elongation\;at\;break=\frac{∆L}{L_{0}}\times 100$$

where Δ*L* is the difference in length of the specimen before and after applying force until the break.

(5) $$Modulus\;at\;break\;\left(\mathrm{MPa}\right)=\frac{Stress\;at\;break\;(N)}{Strain\;at\;break\;(m^{2})}$$

#### 2.4.9. Thermal Properties

Thermal properties were measured by DSC and TGA techniques (METTLER TOLEDO (Columbus, OH, USA), software: STARe version 13). Films were cut into small sizes and weighed to 6 mg before placing them in the DSC sample pan. The test conditions were: (1) first heating from −50 °C to 180 °C, (2) cooling from 180 °C to 0 °C, and (3) second heating from 0 °C to 180 °C, with a heating rate of 10 °C/min under nitrogen flow. The degree of crystallinity was also calculated by following Equation (6).

(6) $$\%X_{c}=\frac{\left(∆H_{m}\right)}{∆H_{m}^{*}}\times 100\%$$

where ∆*H_m_* is the melt enthalpy and $∆H_{m}^{*}$ is the PBS theory enthalpy of 110.3 J/g [37]. 

#### 2.4.10. Decomposition Temperature

The decomposition temperature of the films was characterized by thermal gravimetric analysis (TGA, METTLER TOLEDO TGA/DSC, software: STARe version 13). The sample was cut into small sizes and approximately 6 mg was placed in the aluminum pan. The ramp temperature was controlled at 10 °C/min from 25 °C to 800 °C under a nitrogen atmosphere. The results are presented in the form of a TGA thermogram. 

## 3. Results and Discussion

The synergistic effects of two different Thai natural colorants, roselle calyx (RS) and sappan heartwood (SP) extracts, on the physical, chemical, and mechanical properties of biodegradable films based on poly(butylene succinate) (PBS) were explored for the first time. Films of PBS blended with different concentrations of either RS or SP (0.1, 0.2, or 0.3% wt) were observed and are shown with the abbreviations of 0.1 RS, 0.2 RS, 0.3 RS, 0.1 SP, 0.2 SP, and 0.3 SP. 

**Figure 1:**
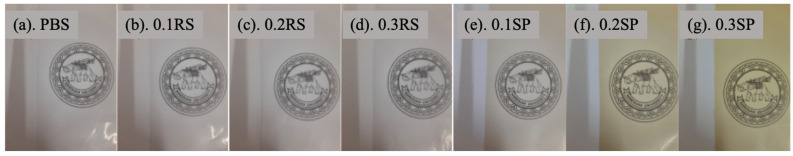
Optical appearances of films of: (**a**) neat PBS and PBS blended with: (**b**) 0.1% wt RS, (**c**) 0.2% wt RS, (**d**) 0.3% wt RS, (**e**) 0.1% wt SP, (**f**) 0.2% wt SP, and (**g**). 0.3% wt SP.

It was found that the concentrations of RS and SP affected the optical appearances of the films, in which the color slightly changed from clear (PBS, Figure 1a) to a light pink–purple tone (PBS/RS) (Figure 1b–d), and to a yellow tone (PBS/SP) (Figure 1e–g), but all films were transparent.

### 3.1. Color Parameter Index

Apart from the optical appearances observed, the color parameter indexes of all films were also analyzed and are shown as the values of “*L*” (lightness (100) to darkness (0), “*a*” (greenness (−a) to redness (+a), “*b*” (blueness (−b) to yellowness (+b) and “∆*E*” (the different color) (Table 1). PBS films without natural colorants had high transparency, while the PBS/RS and PBS/SP films still showed high transparency but with variations in color. The redness values of PBS/RS films were slightly increased, while the yellowness values were slightly decreased, indicating a red tone of the PBS/RS films. The PBS/SP films displayed a noticeable yellow to orange tone in their optical appearance. Increased concentrations of colorant extracts resulted in more pronounced red (RS) and yellow (SP) colors in the films, and more color intensity with lower L values. 

The ∆*E* values of the PBS/RS and PBS/SP films were higher compared to the pure PBS film. This can be attributed to the presence of polyphenols in the natural colorants, which enhance the light protection properties of the films [38]. 

Furthermore, all films underwent an accelerated aging test for a duration of 2 months. The results showed a reduction in shading colors, specifically in terms of lightness, redness, and yellowness. The observed alterations in the color in the films post-aging may potentially be a consequence of the inherent decomposition of distinct pigment constituents, notably the anthocyanins in RS extracts and the neoflavonoids present in SP extracts. 

More precisely, the primary anthocyanins identified in RS extracts—delphinidin-3-sambubioside and cyanidin-3-sambubioside—manifest a finite stability, rendering them prone to modifications under the influence of various environmental stimuli such as temperature fluctuations, oxygen exposure, photonic interactions, and chronological progression [39]. The probable degradation pathway has been hypothesized, implicating the disruption of the B-ring, subsequently leading to the formation of a transient delphinidin chalcone intermediate, a phenomenon discussed in the literature [40]. 

Furthermore, it has been documented that brazilin, which is found in SP extracts, exhibits a high sensitivity to oxidative changes when directly exposed to air, heat, and light. This oxidative process converts its hydroxyl groups into carboxyl groups, resulting in a structural transformation and the formation of a distinct colored compound known as brazilein [41].

In addition, it has been shown that Δ*E* values between 1.0 and 2.0 are considered noticeable only to experienced observers, and a clear difference in color is noticed when a Δ*E* value is greater than 3.5 [42]. The Δ*E* value of the PBS/RS films was less than 2.0, whereas that of the PBS/SP films was above 3.5. This implies stability of the PBS/RS film, as the color change of the RS extract in the PBS film cannot be observed. This change can be attributed to moisture, which has the ability to attract and degrade the chemical structures of the colorants through oxidation. The oxidation reactions of the double bonds present in RS and SP may contribute to color instability [43].

### 3.2. Light Barrier Properties

Light barrier properties were tested by UV–Vis spectrophotometry at different wavelengths of 200–800 nm. Four wavelength regions (UV-C (280 nm), UV-B (315 nm), UV-A (400 nm), and the visible region (700 nm)) were used to test for %T (Table 2.). The PBS film showed high %T for visible light, followed by UV-A, UV-B, and UV-C, respectively. For all other PBS/RS and PBS/SP films, the percent transmittance (%T) values were found to be similar to that of the pure PBS film. However, higher concentrations of RS and SP in the films resulted in lower %T values. Notably, when subjected to UV-C radiation at 280 nm, all films exhibited the lowest %T values. This can be attributed to the absorption of light by sensitive chromophores in RS that have an aromatic ring with a double-bond chemical structure, leading to reduced transmittance. A high content of SP reduced the %T of visible light in the PBS/SP films due to the attribution of the neoflavonoid as brazilin in sappan [44,45]. 

**Figure 2:**
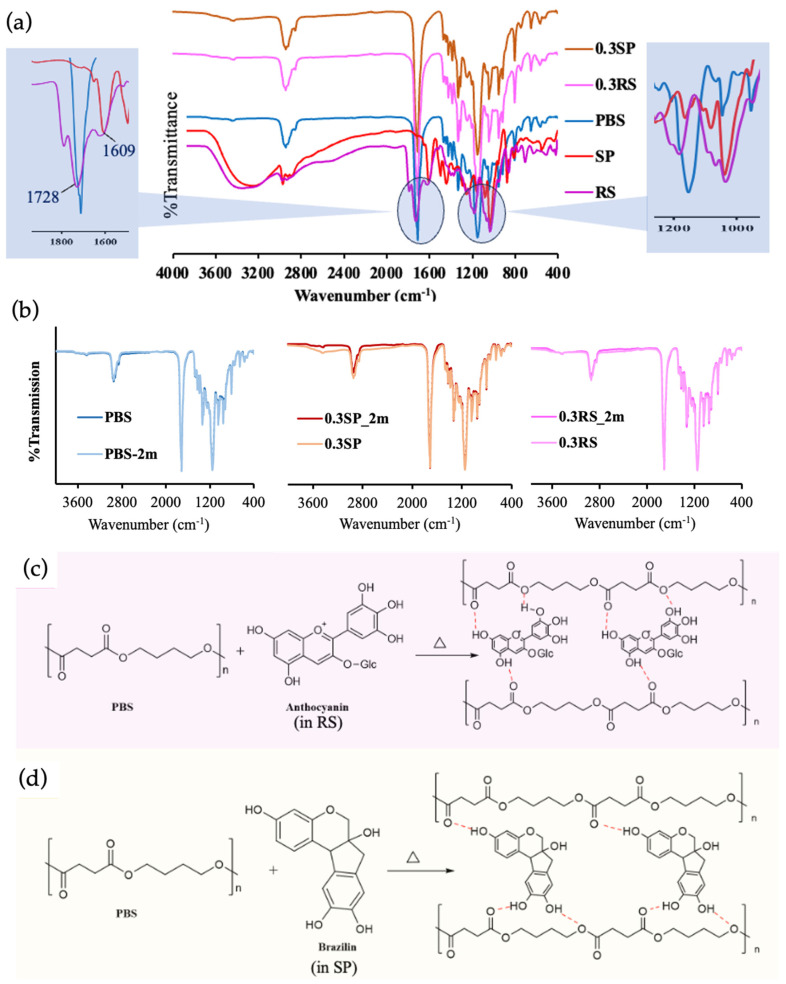
FTIR spectra (FTIR−ATR mode) of PBS, RS, SP, and their films at the origin time (**a**) and after 2 months of accelerated aging (**b**), possible chemical reactions between PBS and anthocyanin in RS (**c**), and PBS and brazilin in SP (**d**).

Following the 2-month aging test, the light transmission properties of PBS and PBS/RS films showed an increasing trend, likely attributed to the oxidation effect [24]. In contrast, the light transmission properties of the PBS/SP films exhibited a slight decrease, resulting in improved light barrier properties. This can be attributed to the presence of polyphenol compounds, including neoflavonoids found in SP, which have the ability to form hydrogen bond interactions within the polymer chain, thus enhancing the light-blocking capabilities of the films. An example of a polyphenol compound found in the SP extract is brazilin, which contains hydroxyl functional groups capable of engaging in interactions with the carbonyl groups found in PBS, as seen in Figure 2c.

### 3.3. Water Vapor Transmission Rate (WVTR)

**Figure 3:**
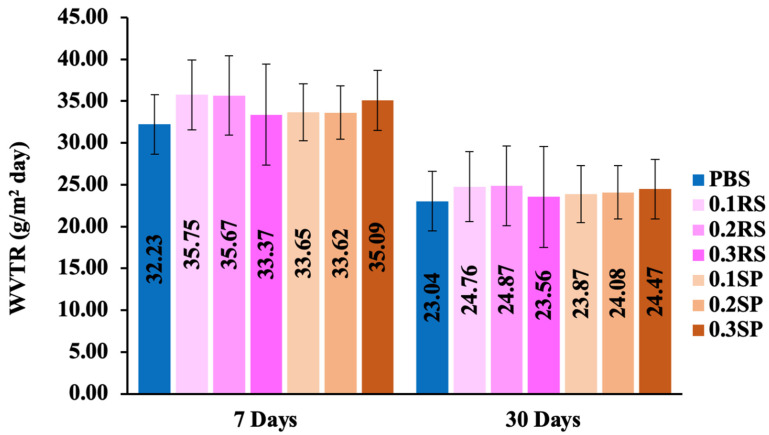
Water vapor transmission rate (*WVTR*) of PBS, PBS/RS, and PBS/SP films at different concentrations of colorants at 0.1, 0.2, and 0.3% wt at days 7 and 30.

*WVTR* is an important property of film packaging during the shelf-life period. Hence, *WVTR* testing was conducted on PBS, PBS/RS, and PBS/SP films. The results revealed that the incorporation of RS and SP extracts in the PBS films slightly enhanced their *WVTR* compared to neat PBS films, both after 7 and 30 days of testing (Figure 3). This enhancement can be attributed to the increased mobility of the polymer chains and a less compact film structure, facilitating faster transfer of water and moisture [46]. Interestingly, when comparing the average *WVTR* values over a span of 7 days to those of 30 days, all films exhibited a notable decline, with *p*-values < 0.01 for every sample. This decrease can be attributed to the potential increased absorption of water into the film matrix over the testing period, leading to lower *WVTR* values.

### 3.4. Chemical Properties

The chemical functional groups of PBS, RS, SP, and their films at the origin time (Figure 3a) and after 2 months of accelerated aging (Figure 3b) were characterized by FTIR, and the possible reactions between PBS and either anthocyanins (the phenolic compounds of the flavonoid class in RS) or brazilin (the flavonoids in SP) are presented (Figure 3c,d, respectively). Generally, RS and SP exhibit colors in the red spectrum, but their specific colors can vary depending on the transformations occurring in their chemical structures. Anthocyanin and brazilin mainly contain aromatic, carbonyl, and hydroxyl groups [47,48]. From the FT-IR spectra, the broad bands at 3354 and 3255 cm^−1^ are attributed to the O-H stretching with hydrogen bonding of RS and SP, respectively, while the bands at 2976 and 2972 cm^−1^ indicate the CH_3_ and CH_2_ stretching of both extracts. Bands at 1728 and 1609 cm^−1^ suggest the presence of C=O in the flavonoid skeleton, and sharp bands at 1035 and 1038 cm^−1^ suggest the presence of the C-O group, which is related to the characteristics of anthocyanin (in RS) and brazilin (in SP) [49]. 

The characteristic bands of PBS show at 1750 cm^−1^, related to the C=O of the ester group, and at 1180 cm^−1^, corresponding to the C-O-C of the PBS backbone [50]. The FT-IR bands of the PBS/RS and PBS/SP films were simply the spectrum of each component with small changes in band intensity at -OH stretching with hydrogen bonding. 

Furthermore, the FT-IR bands for all films, both at the origin time and after 2 months of accelerated aging, remained consistent. This consistency is attributed to the unchanged chemical structures of their material’s functional groups during the observed period.

However, possible hydrogen bonds between the -OH groups of anthocyanin (in RS) and brazilin (in SP) with the -C=O and C-O-C of PBS are expected to occur.

### 3.5. Tensile Properties

**Figure 4:**
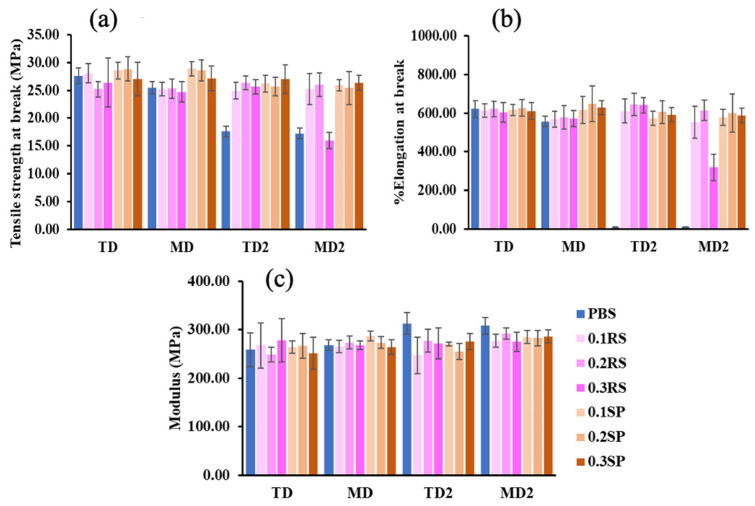
Tensile properties of PBS, PBS/RS, and PBS/SP films in the TD and MD directions at the origin time (TD, MD) and after 2 months of accelerated aging (TD2, MD2): (**a**) tensile strength at break, (**b**) % elongation at break, (**c**) modulus.

The tensile properties, including tensile strength at break, elongation at break, and the modulus, of all films were evaluated in two directions: the machine direction (MD) and transverse direction (TD), both at the origin time and after 2 months of accelerated aging. The results are presented in Figure 4. In Figure 4a, the tensile strength of the neat PBS film was 27.63 (TD) and 25.44 (MD) MPa. However, a significant decrease in tensile strength was observed for the PBS film after undergoing the 2-month accelerated aging process. The aging process allows time for hydrolysis reactions to occur in the aged PBS, resulting in the decomposition of PBS chains into smaller units [51]. 

All films of PBS/RS and PBS/SP showed similar values of the tensile strength at break to neat PBS. Figure 4b demonstrates that the percent elongation at break of all films, including PBS, exhibited similar values initially. Again, there was a notable decrease in the percent elongation of the neat PBS film after the 2-month accelerated aging period. 

Interestingly, films that incorporated RS or SP extracts continued to exhibit a high percent elongation (around 600%) even after a 2-month accelerated aging period. PBS can degrade over time due to various environmental factors such as heat, oxygen, UV radiation, and moisture [51]. The molecular interactions between RS, SP, and PBS, particularly hydrogen bonding, enhance the composite’s resistance to these environmental factors, making it more robust than pure PBS [52]. This likely accounts for the maintained percent elongation in films with RS and SP, even after the 2-month accelerated aging period. 

Regarding Figure 4c, the modulus values of all films appeared to be similar, except for the PBS films after the 2-month accelerated aging, which exhibited higher values. This can be attributed to their lower percent elongation at break compared to the other films.

In this framework, the design of more sustainable and durable composites using PBS as the matrix and non-lignified plant fibers such as hemp and flax could also lead to an improvement of the interfacial forces between the matrix and the fillers, inclusive of RS and SP based on their polar natures. The synergistic impact of non-lignified plant fibers in combination with RS and SP is expected to improve the performance exerted by PBS composites.

### 3.6. Fractured Surface Morphology

**Figure 5:**
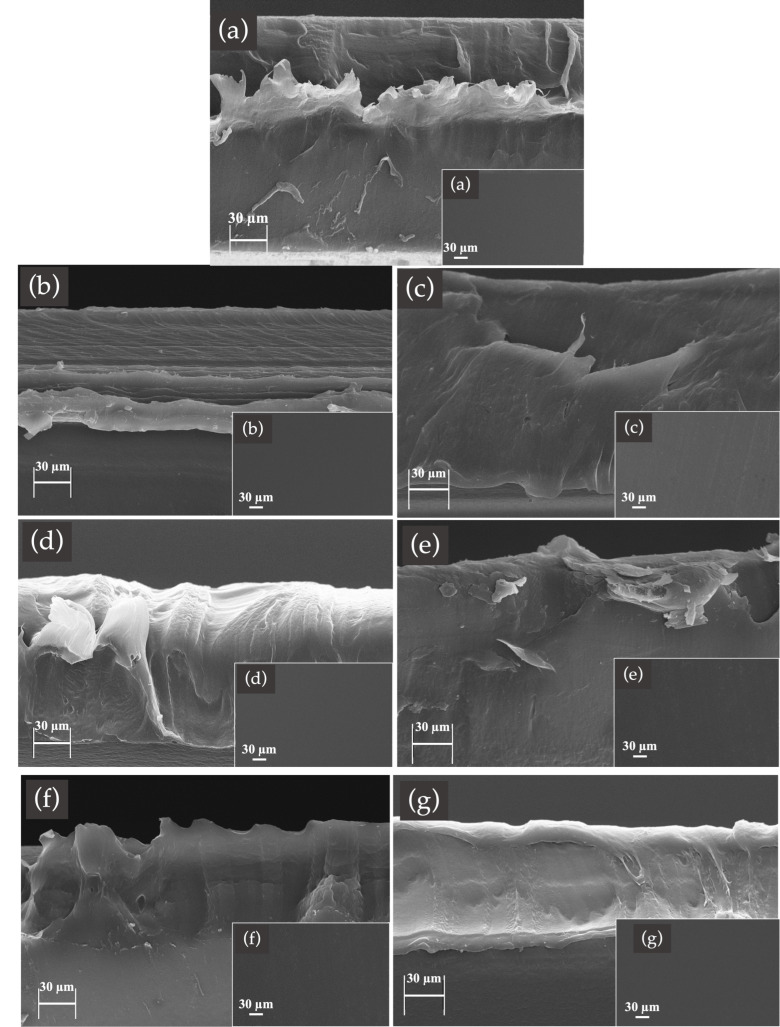
Fractured surface morphology of: (**a**) PBS, (**b**) 0.1 RS, (**c**) 0.2 RS, (**d**) 0.3 RS, (**e**) 0.1 SP, (**f**) 0.2 SP, and (**g**) 0.3 SP at a magnification of 500×; inset images are the surface morphology of the films.

The morphology of the fractured surfaces (in liquid nitrogen) of the neat PBS film and the PBS blended with either RS or SP extracts (at 0.1, 0.2, and 0.3% wt) were observed (Figure 5). All films exhibited a homogeneous distribution of PBS and colorant extracts with no phase separation, roughness, or aggregation of RS and SP. The incorporation of varying concentrations of RS and SP into the PBS films did not notably influence the phase morphology of the films, a phenomenon evidenced by the homogeneity observed at the surfaces of all of the analyzed films (inset images). The results from the SEM images are in agreement with the results from the % elongation (Figure 4b).

### 3.7. Crystallinity

**Figure 6:**
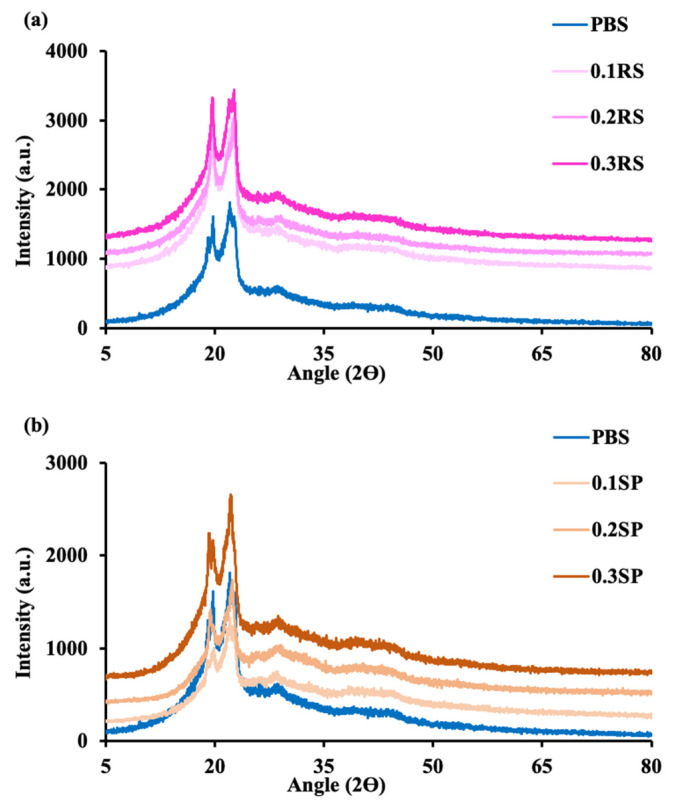
XRD patterns of: (**a**) PBS and PBS/RS and (**b**) PBS and PBS/SP films at different concentrations of colorants at 0.1, 0.2 and 0.3% wt.

The crystallinity characteristics of the films were measured by X-ray diffraction, and the XRD patterns are shown in Figure 6. The results indicate the different diffraction peaks, which are related to the crystal unit cell of PBS. The PBS crystal unit cell is a monoclinic pattern that has diffraction peaks at (020), (021), and (110) with angles of approximately 19.5°, 21.5°, and 22.5°, respectively [53,54]. Incorporating RS and SP extracts into the films had a slight effect on the diffraction angles and peak intensities of the PBS. The PBS/RS and PBS/SP films exhibited slightly lower peak intensities compared to pure PBS. This result agreed with the results from the % elongation (Figure 4b) that there are no significant differences in structure between the PBS films and the PBS/RS and PBS/SP films.

### 3.8. Thermal Properties

**Figure 7:**
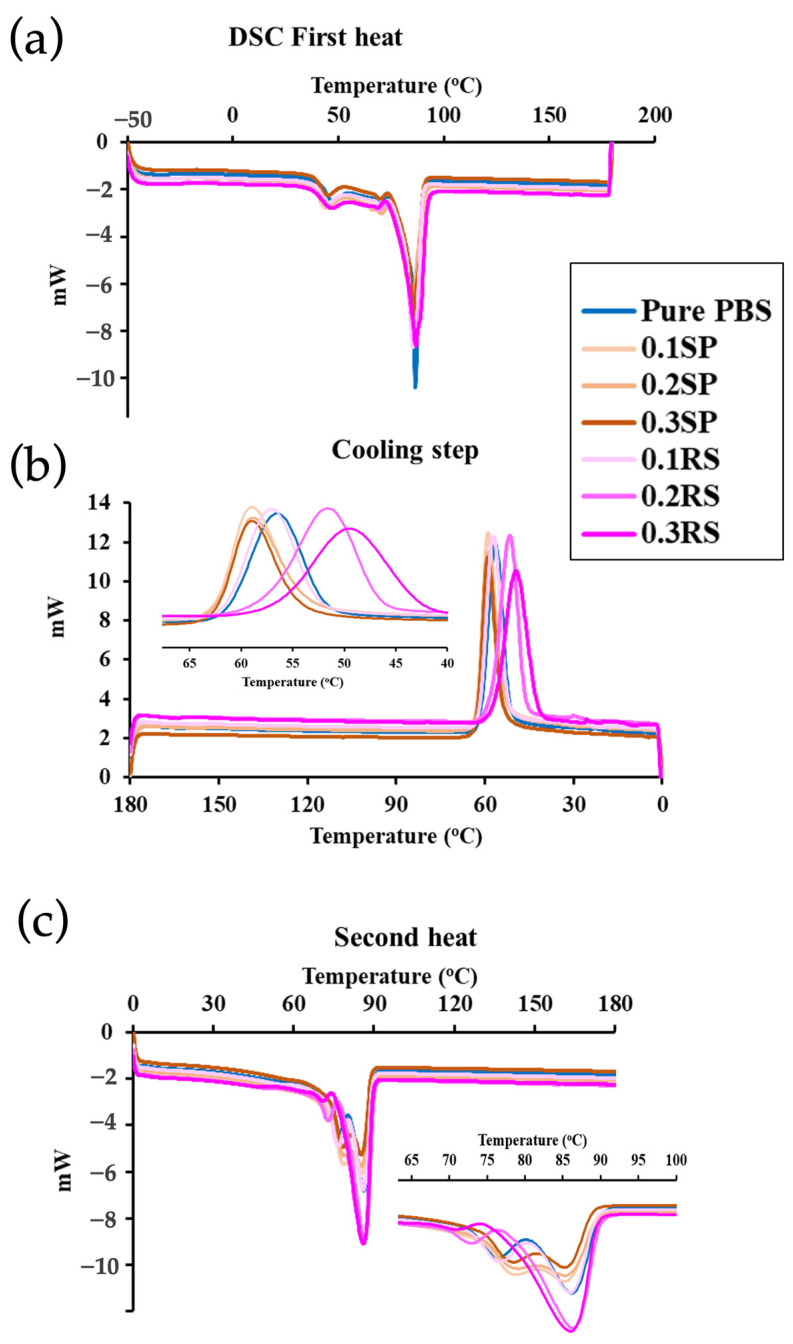
DSC thermogram of PBS, PBS/RS, and PBS/SP films at different concentrations of colorants at 0.1, 0.2, and 0.3% wt.: (**a**) First heating, (**b**) Cooling step, (**c**) Second heating, with a heating and cooling rate of 20 °C/min.

The thermal properties and crystallinity (calculated from the DSC) of all films were also observed as shown in Table 3 and Figure 7. From the first heat cycle, the glass transition temperature (T_g_) of PBS was at 47.10 °C, and the lowest T_g_ of all films was observed when RS and SP were added. As seen, the decrease in T_g_ was influenced by the natural colorant chemical structures that provided plasticizer and compatibilizer effects, improving the free volume of the PBS structure [55]. 

After cooling and the second heating cycle, a significant increase in melt enthalpy (∆*H_m_*) was observed in the films incorporating RS and SP compared to neat PBS. This increase corresponded to an enhancement in the percentage of crystallinity (%X_c_). This finding is consistent with the XRD patterns and can be attributed to the presence of phenolic compounds in RS and SP, which act as nucleating agents. These compounds promote hydrogen bonding with the PBS chains, limiting their mobility and facilitating re-crystallization. 

The observed increase in the percentage of crystallinity can be attributed to the rearrangement of polymer chains, aided by the hydroxyl groups of the phenolic compounds in RS and SP, which generate hydrogen bonding and hinder chain mobility [56].

### 3.9. Thermal Decomposition

**Figure 8:**
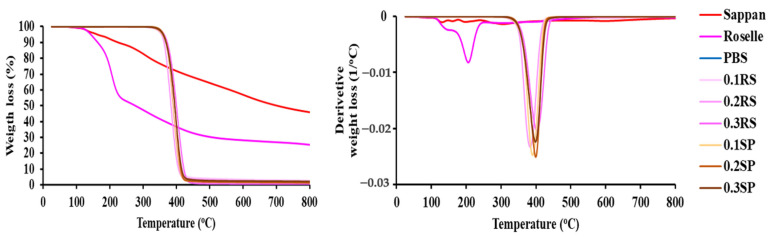
TGA thermogram of dried extracts (sappan and roselle) and PBS, PBS/RS, and PBS/SP films containing different concentrations (0.1, 0.2, and 0.3% wt) of colorant.

Thermogram decomposition (TGA/DTG curves) of PBS, RS, SP, and their films were also studied (Figure 8). All samples were dried before testing to remove all solvents and moisture. The primary decomposition of RS showed a peak at 200 °C related to the decomposition of anthocyanin components, which was a chlorogenic acid composition weight loss of 46.14% [57]. SP decomposed at 130.95 °C (4.34% weight loss), 197.87 °C (10.35% weight loss), and above 300 °C (85.31% weight loss), which are related to the decomposition temperatures of flavonoid compounds in the SP extract. The neat PBS film decomposed at 405.30 °C for 95.37% weight loss. However, the PBS/RS and PBS/SP films showed a slight increase in decomposition temperature due to the stronger hydrogen bonding generated between PBS and either RS or SP. The concentration of RS and SP extracts did not have a substantial effect on the thermal decomposition behavior of the films.

## 4. Conclusions

In this study, films based on PBS were successfully prepared using commonly employed industrial methods, incorporating two distinct Thai natural colorants: roselle calyx (RS) and sappan heartwood (SP) extracts. The addition of RS and SP extracts resulted in a slight improvement in the tensile elongation of the films compared to neat PBS, while also demonstrating excellent UV–Vis light barrier properties. Thermal property and crystallinity analyses indicated that the presence of anthocyanin (in RS) and brazilin (in SP) contributed to the enhancement of film crystallinity and improved compatibility with PBS. Interestingly, no significant differences were observed in the physical or mechanical properties of the films incorporating RS and SP extracts. These findings highlight the potential of PBS/RS and PBS/SP films for applications in film packaging that prioritize biodegradability and the non-toxic release of color molecules. The combination of enhanced tensile elongation, UV–Vis light barrier properties, improved crystallinity, and compatibility with PBS positions these films as promising alternatives in sustainable packaging solutions. For further work, the assessment of the effect of hybrid mixtures of RS and SP colorants on the performance of composites made of PBS should be interesting to establish as another choice for sustainable packaging, especially for health care products.

## Figures and Tables

**Table 1:** Color parameter index of biodegradable packaging films incorporating natural colorants before and after an accelerated aging test.

Sample	Origin				2 Months			
	L*	a*	b*	∆E*	L*	a*	b*	∆E*
(a). PBS	89.28	−0.86	−4.18	Ref	89.44	−1.06	−4.50	0.41
	±0.08	±0.05	±0.13		±0.21	±0.05	±0.07	
(b). 0.1 RS	87.54	−0.20	−4.72	1.94	89.14	−1.00	−3.72	0.50
	±0.29	±0.00	±0.15		±0.26	±0.00	±0.19	
(c). 0.2 RS	87.26	0.46	−5.10	2.58	89.06	−0.90	−3.52	0.70
	±0.09	±0.05	±0.07		±0.15	±0.00	±0.16	
(d). 0.3 RS	85.58	1.86	−5.20	4.70	88.70	−0.70	−2.90	1.41
	±0.45	±0.15	±0.10		±0.37	±0.00	±0.12	
(e). 0.1 SP	84.20	0.36	16.54	21.37	84.32	1.16	12.66	3.96
	±0.16	±0.05	±0.42		±0.38	±0.18	±0.71	
(f). 0.2 SP	82.68	0.46	26.58	31.49	81.32	2.04	21.86	5.16
	±0.22	±0.09	±0.69		±1.37	±0.63	±2.40	
(g). 0.3 SP	82.92	−0.24	29.90	34.67	80.22	2.20	26.60	4.91
	±0.29	±0.11	±1.12		±1.23	±0.55	±2.51	

Note: the reference is the origin values of the same films for calculating the ΔE* values after 2 months.

**Table 2:** UV–VIS light barrier properties of the films.

Samples	%T at Origin				%T at 2 Months			
	280 nm (UV-C)	315 nm (UV-B)	400 nm (UV-A)	700 nm (Visible)	280 nm (UV-C)	315 nm (UV-B)	400 nm (UV-A)	700 nm (Visible)
PBS	4.33 ± 1.15	11.00 ± 1.73	28.00 ± 2.65	65.33 ± 2.08	5.00 ± 1.00	11.67 ± 1.53	29.67 ± 2.08	66.33 ± 1.53
0.1 RS	4.33 ± 0.58	11.00 ± 1.00	29.67 ± 1.15	66.33 ± 0.58	5.67 ± 1.53	13.00 ± 1.73	30.67 ± 3.21	66.33 ± 1.53
0.2 RS	2.67 ± 0.58	8.00 ± 1.00	24.00 ± 1.00	61.67 ± 1.15	4.33 ± 1.15	9.67 ± 2.31	26.33 ± 2.89	62.33 ± 2.08
0.3 RS	2.33 ± 0.58	7.00 ± 1.00	21.67 ± 2.08	59.33 ± 1.53	3.33 ± 1.15	8.33 ± 2.89	25.00 ± 4.36	61.67 ± 3.21
0.1 SP	3.33 ± 1.15	10.00 ± 1.73	25.00 ± 2.65	63.67 ± 1.53	2.67 ± 1.15	8.00 ± 1.73	22.67 ± 2.89	60.67 ± 2.08
0.2 SP	2.33 ± 1.15	8.67 ± 2.31	22.67 ± 3.21	62.33 ± 2.08	1.67 ± 0.58	6.67 ± 0.58	20.33 ± 1.15	61.00 ± 1.00
0.3 SP	1.33 ± 0.58	6.33 ± 1.53	18.00 ± 1.73	60.00 ± 1.00	1.67 ± 0.58	7.67 ± 1.53	21.00 ± 2.65	61.00 ± 1.00

**Table 3:** DSC results of PBS, PBS/RS, and PBS/SP films at different concentrations of RS and SP (0.1, 0.2, and 0.3% wt).

Profile at Peak						
Samples	T_g_ (°C)	T_c_ (°C)	∆H_c_ (Jg^−1^)	T_m_ (°C)	∆*H_m_* (Jg^−1^)	%X_c_
**PBS**	47.10	56.51	45.08	86.27	16.61	15.06 ± 4.1
**0.1 RS**	45.27	57.19	44.43	86.11	39.78	36.07 ± 3.2
**0.2 RS**	46.26	51.68	47.32	86.37	31.08	28.18 ± 6.0
**0.3 RS**	46.09	49.62	49.32	86.27	37.28	33.80 ± 4.4
**0.1 SP**	43.76	59.03	41.09	85.30	47.47	43.04 ± 3.1
**0.2 SP**	44.93	58.83	49.32	86.03	37.28	33.80 ± 5.4
**0.3 SP**	44.77	58.99	41.35	85.16	46.04	41.74 ± 3.0

## Data Availability

The raw/processed data required to reproduce these findings cannot be shared at this time as the data also forms part of an ongoing study.
